# Comparison of the effects of rumen-protected and unprotected *L*-leucine on fermentation parameters, bacterial composition, and amino acids metabolism in *in vitro* rumen batch cultures

**DOI:** 10.3389/fmicb.2023.1282767

**Published:** 2023-11-23

**Authors:** Jishan An, Weijun Shen, Hu Liu, Chen Yang, Kemeng Chen, Qiongwen Yuan, Zhiqing Li, Dingfu Xiao, Zuo Wang, Xinyi Lan, Lei Liu, Fachun Wan

**Affiliations:** ^1^College of Animal Science and Technology, Hunan Agricultural University, Changsha, Hunan, China; ^2^College of Veterinary Medicine, Hunan Agricultural University, Changsha, Hunan, China

**Keywords:** *L-*leucine, beef cattle, *in vitro* technique, rumen protection rate, rumen fermentation, bacterial community

## Abstract

This study was conducted to compare the effects of rumen-protected (RP-Leu) and unprotected *L*-leucine (RU-Leu) on the fermentation parameters, bacterial composition, and amino acid metabolism *in vitro* rumen batch incubation. The 5.00 g RP-Leu or RU-Leu products were incubated *in situ* in the rumen of four beef cattle (*Bos taurus*) and removed after 0, 2, 4, 6, 12, 16, and 24 h to determine the rumen protection rate. In *in vitro* incubation, both RP-Leu and RU-Leu were supplemented 1.5 mmol/bottle (*L*-leucine HCl), and incubated after 0, 2, 4, 6, 8, 12, and 16 h to measure gas production (GP), nutrient degradability, fermentation parameters, bacterial composition, and amino acids metabolism. Results from both *in vitro* and *in situ* experiments confirmed that the rumen protection rate was greater (*p* < 0.01) in RP-Leu than in RU-Leu, whereas the latter was slow (*p* < 0.05) degraded within incubation 8 h. Free leucine from RP-Leu and RU-Leu reached a peak at incubation 6 h (*p* < 0.01). RU-Leu supplementation increased (*p* < 0.05) gas production, microbial crude protein, branched-chain AAs, propionate and branched-chain VFAs concentrations, and Shannon and Sobs index in comparison to the control and RP-Leu supplementation. RU-Leu and RP-Leu supplementation decreased (*p* < 0.05) the relative abundance of *Bacteroidota*, which *Firmicutes* increased (*p* < 0.05). Correlation analysis indicated that there are 5 bacteria at the genus level that may be positively correlated with MCP and propionate (*p* < 0.05). Based on the result, we found that RP-Leu was more stable than RU-Leu in rumen fluid, but RU-Leu also does not exhibit rapid degradation by ruminal microbes for a short time. The RU-Leu was more beneficial in terms of regulating rumen fermentation pattern, microbial crude protein synthesis, and branched-chain VFAs production than RP-Leu *in vitro* rumen conditions.

## 1 Introduction

The 3 branched-chain AAs (BCAAs), namely, leucine (Leu), isoleucine (Ile), and valine (Val), are categorized as essential amino acids (EAAs) used by tissues as substrates for protein synthesis and energy generation (Bergen, 2021; Silva et al., 2022), and the modulation of cell signaling pathways (Nie et al., 2018; Xu et al., 2022). Moreover, the BCAAs represent up to 35 and 50% of all EAAs in muscle and milk for most mammals, respectively (Appuhamy et al., 2011; Wu, 2013), while free BCAAs mainly sourced from microbial crude protein and feed undegraded protein in the rumen for ruminants.

Leu, the highest proportion in BCAAs, is the effective amino acid for activating the mTOR signaling pathways and protein synthesis (Zhang et al., 2017; Xu et al., 2022). Besides, Leu is one of the important components in MCP, which is second only to lysine (Kajikawa et al., 2002). At present, due to the characteristic of amino acid degradation by rumen microbes (Tan et al., 2021), the application forms of Leu are rumen-protected (RP-Leu) and unprotected *L*-Leucine (RU-Leu). These studies focused on the activities of small intestinal digestive enzymes and lactation performance for dairy cows and goats, but the results were inconsistent. Few studies have reported that supplementation with rumen-protected BCAAs (Leu: Ile: Val = 4:1:1) did not affect milk protein yield and rumen fermentation (Xu et al., 2022), while Yepes et al. (2019) found that dietary rumen-protected BCAAs supplementation from calving increased the free Leu and Val plasma concentration *in vivo* animal experiments. Whereas others studies have suggested that dietary rumen undegraded leucine could reach comparable leucine physiology function as RP-Leu, such as stimulating the rumen bacterial growth (Yang, 2002), improving the mRNA abundance of BCAAs transport and enzymes (Webb et al., 2020), stimulate the milk and muscle or immune protein synthesis (Lapierre et al., 2002; Appuhamy et al., 2011), but studies on the mechanism of action are limited. Moreover, Hultquist and Casper (2016) have confirmed that dietary supplementation of rumen degradable valine could increase milk yield for late-lactating dairy cows. Compared to *in vivo* animal studies, *in vitro* batch incubation excludes the effect of physiological conditions and is a useful method to investigate actual fluxes of VFA production and MCP synthesis (Wang et al., 2019). Few studies have been performed to investigate the effect of supplementation different ratios of BCAA on rumen fermentation and amino acids metabolism through *in vitro* batch fermentation (Zhang et al., 2013).

At present, previously suggested that the BCAA content in ruminant by-products are closely associated with the free BCAA content of the rumen fluid and blood (Curtis et al., 2018; Boehmer et al., 2020). Microbial crude protein (MCP) in rumen fluid accounts for 50 to 80% of total absorbable protein (Firkins et al., 2007), and conversion between BCAAs and branched-chain volatile fatty acids (BCVFAs) in rumen, thus, the MCP and BCVFAs in rumen fluid may be effectively pathways for increased the free BCAAs concentration in serum (Clark et al., 1992; Kajikawa et al., 2002). Based on the above analysis, we proposed the hypothesis that supplementation *L-*Leu can enhance free leucine levels in plasma for beef cattle by improving rumen protection rate, BCVFA, and MCP synthesis. However, the literature is scarce and limited in terms of reports on the degradation and bioavailability of RU-Leu and RP-Leu in beef cattle. To fill the gap, we conducted *in vitro* batch incubation to investigate and compare the effects of RU-Leu and RP-Leu on rumen fermentation, bacterial composition, and amino acid metabolism.

## 2 Materials and methods

### 2.1 Substrates

The substrates for *in vitro* incubation were total mixed ration (TMR) containing wheat straw and concentrate mixture (4:6), as same as rumen fluid donor beef cattle diet. They were dried at 65°C, ground to pass a 1 mm screen, and stored in air-tight bags, respectively. The chemical composition of the TMR and fermentation substrates of the *in situ* and *in vitro* experiments were determined (Table 1).

**Table 1:** Ingredients and chemical composition of the basal TMR and fermentable substrates of the *in situ* and *in vitro* experiments.

Items	Contents
Ingredients (%)	
Concentrate mixture^1^	60.00
Wheat straw	40.00
Chemical composition^2^	
DM, g/kg as fed	912
CP, g/kg DM^3^	121
NDF, g/kg DM	472
ADF, g/kg DM	244
EE, g/kg DM	35.6
Ca, g/kg DM	8.39
P, g/kg DM	5.28
Leu, g/kg DM	9.75

DM, dry matter; CP, crude protein; NDF, neutral detergent fiber; ADF, acid detergent fiber; EE, ether extract; Ca, calcium; P, phosphorus; Leu, Leucine. ^1^Concentrate mixture: corn, soybean meal, expanded soybean, corn distiller’s grains, salt, limestone, premix. ^2^Chemical composition: all nutrient levels were the measured values. ^3^Calculated as nitrogen × 6.25.

The additives for *in vitro* incubation were rumen-unprotected *L*-leucine (RU-Leu) and rumen-protected *L*-leucine (RP-Leu). The RU-Leu products (food-grade; purity, ≥99.5%), were purchased from Hebei Huayang Biotechnology Co., Ltd. (batch no: 20220519; Hengshui, China). The quality of *L*-leucine met the national standards in China (GB 29938/ USP30). The RP-Leu products (feed-grade; purity, ≥75%; microencapsulation; particle size, 5 mesh sieve through, NLT90%; moisture, 1.1%; appearance, straw yellow granule), which were purchased from Hangzhou King Techina Feed Co., Ltd. (batch no: 20220428; Hangzhou, China). Additionally, according to the certificate of manufacturers analysis, the rumen-protected rate of RP-Leu is 85% in the simulated rumen conditions for 12 h, and the release rate is 89.4% in the simulated intestinal conditions for 12 h.

### 2.2 *In situ* incubation

Four Xiangxi yellow cattle (*Bos taurus*, local breeds, Hunan, China) fitted with permanent ruminal cannulas were fed a total mixed ration containing wheat straw and concentrate mixture (4, 6; Table 1) and had free access to water. The *in situ* incubation was performed by described in detail by Griffith et al. (2017). According to the “all in / gradual out” schedule, all of the nylon bags (pore size: 50 μm, size: 10 cm × 7 cm) with 5.00 g of the RP-Leu or RU-Leu products were tied to the end of a 40 cm polyester mesh tube and then put into the ventral sac of the rumen through a ruminal cannula after morning feeding 1 h. Two nylon bags were collected from the cannula of each cattle at 0, 2, 4, 6, 12, 16, and 24 h of incubation, respectively, with 8 replicates per time point (*n* = 8), immediately submerged in the ice water to stop microbial activity, rinsed with running water until the water was clear. All RU-Leu and RP-Leu samples were oven-dried at 50°C for 48 h and then weighed.

### 2.3 *In vitro* incubation

Rumen contents were collected via the rumen cannula from three fistulated Xiangxi yellow cattle before morning feeding. The cattle were fed a total mixed ration containing wheat straw and concentrate mixture (4, 6; Table 1) and had free access to water (Yáñez-Ruiz et al., 2016). The rumen contents were filtered through a 4-layer cheesecloth individually and then were equally mixed. Fresh rumen fluid was then mixed with McDougall’s buffer (McDougall, 1948) at a ratio of 1:2 (vol/vol) to prepare the buffered rumen fluid. All the procedures were conducted under an anaerobic condition with a stream of CO_2_. About 1.0000 *g* of fermentation substrate was weighed into a 200 mL fermentation bottle (Huake Labware Co. Ltd., Shanghai, China), and supplemented with 0 mg of additives (Control), 12 mg RU-Leu (1.5 mmol/bottle *L*-leucine HCl) (RU-Leu), and 16 mg RP-Leu (1.5 mmol/bottle *L*-leucine HCl) (RP-Leu), and incubated with 60 mL of buffered rumen fluid under a steam incubator of CO_2_ at 39.5°C and 55 rpm. The *in vitro* fermentation was performed in the automated batch incubation devices described in detail in Cornou et al. (2013). The cumulative gas production (GP) of each bottle was measured with a pressure transducer (SW-512C; Senwei Electronics Co. Ltd., Dongguan, China) (Mauricio et al., 1999). Each run contained six bottles per treatment (*n* = 6) and was repeated three times on different days and donor cattle so that each treatment was conducted in triplicate.

### 2.4 Procedures and sample collection

For *in situ* incubation experiments. At 0, 2, 4, 6, 12, 16, and 24 h of incubation, RU-Leu and RP-Leu product residues were collected for the determination of rumen protection rates in the rumen. The dynamic rumen protection rate was calculated by the formula: [P = (M_1_-M_2_) /M_1_*100%], where P is the degradation rate of leucine products at time *X*. M_1_ is sample mass; M_2_ is residue mass. Rumen protection rate (%): 100 - P.

For *in vitro* incubation experiments. The *in vitro* incubation was stopped at 0, 2, 4, 6, 8, 12, and 16 h, respectively, and then sampling. About 10 mL of samples were collected and immediately measured for pH values (Seven2Go; Mettler Toledo Technology Co. Ltd., Shanghai, China). About 3 mL of liquid samples were collected, immediately transferred into liquid nitrogen, and stored at –80°C for microbial DNA extraction (Liu et al., 2021). About 5 mL of liquid samples without visible particles were collected from each bottle and centrifuged at 15,000 *g* for 10 min at 4°C, and then 1.5 mL of supernatants were transferred into tubes, acidified with 0.15 mL of 25% (w/v) metaphosphoric acid and stored at −20°C overnight, and subsequently analyzed the volatile fatty acids (VFA) and ammonia (NH_3_-N) concentration of the supernatants.

#### 2.4.1 Fermentable substrates analysis

The dry matter (DM), crude protein (CP) (N × 6.25), and ether extract (EE) contents of the TMR diet and fermentable substrates samples were determined following the procedures of AOAC (1995). Neutral detergent fiber (NDF, assayed with a heat-stable α-amylase and expressed inclusive of residual ash) and acid detergent fiber (ADF) contents in the TMR diet and fermentable substrates were analyzed according to the methods described by Van Soest et al. (1991). The calcium (Ca) and phosphorus (P) contents in the TMR diet were assessed as described previously (Urbaityte et al., 2009; Wang et al., 2020). The amino acids in the fermentable substrates were determined by the microwave hydrolysis method. Briefly, the substrate sample was acid hydrolysis with 6 mol/L HCl at 110°C for 24 h, the hydrolysate was filtered after cooling to ambient temperature, and then transferred into a 50 mL volumetric flask and brought to the volume with ultrapure water. The amino acid concentration was determined using an automatic amino acid analyzer (L-8900, Hitachi Technologies, Inc., Tokyo, Japan).

#### 2.4.2 Rumen fermentation parameters

The VFAs concentration of rumen fluids was determined by gas chromatography (GC) with a capillary column (AT-FFAP: 30 m × 0.32 mm × 0.5 μm) using an Agilent 7890B system (Agilent Technologies, Santa Clara, CA, USA) following the method of Wang et al. (2016). Ammonia (NH_3_) -N concentration was analyzed using a spectrometer (SpectraMax M5, Molecular Devices, San Jose, United States) at an absorbance of 630 nm, following Liu et al. (2022). The microbial crude protein (MCP) concentration of the supernatants was quantified by using Lowry’s assay described by Makkar et al. (1982). Briefly, aliquots of 5 mL of fermentation liquid were centrifuged at 12,000 r/min for 20 min at 4°C, then the precipitates were washed twice with distilled water, the final volume was made to 2 mL with distilled water and vortexed for 1 min, 1 mL of 2 mol/L NaOH was added into 1 mL of the bacterial solution, and cooled after heated in a 95°C water bath for 10 min, the supernatant was collected after centrifugation at 10,000 r/min for 10 min at 4°C. The 1 mL supernatant and 1.5 mL of 0.833 mol/L HCl were mixed, then microbial protein concentration was determined using Bicinchoninic acid Protein Assay Kit (Cat No. MA0082-2, Meilun biotechnology Co., Ltd. Dalian, China), the operations were conducted strictly according to the instructions.

The free amino acids (FAAs) of the fermentation liquid were determined by Hassan et al. (2021). Briefly, the fermentation liquid was centrifuged at 12,000 r/min for 15 min at 4°C, an aliquot of the supernatant was mixed (1:1) with a 10% trichloroacetic solution and vortexed for 1 min, the supernatant was collected after centrifugation at 12,000 r/min for 15 min at 4°C and then filtered with a 0.22 μm filter membrane transferred into an autosampler vial. The FAAs were determined using an automatic amino acid analyzer (L-8900, Hitachi Technologies, Inc., Tokyo, Japan).

#### 2.4.3 DNA extraction, 16S rRNA gene amplification, and sequencing

The total DNA in the fermentation fluid sample was extracted using a kit (Omega Bio-Tek, Norcross, USA), and the concentration and purity of DNA were tested using Nano ⁃ Drop2000. The V3-V4 variable region was amplified by PCR using 338F (5’-ACTCCTACGGGAGGCAGCAGCAG-3′) and 806R (5’-GGACTACH-VGGGTWTCTAAT-3′) primers. PCR products were recovered, purified, eluted, and detected. Quantification was performed using Quanti Fluor TM-ST (Promega, USA). Then, Trimmomatic software was used to detect the quality of the original sequencing sequence, and FLASH software was used to splice it. Finally, the PE300 library was constructed based on the Illumina MiSeq platform. The sequences were clustered by operational taxon (OTU) according to 97% similarity, and single sequences and chimeras were removed. The sequences were clustered by the I-Sanger cloud Platform database^1^ annotates each sequence for species classification.

The α-diversity, including Sobs, Shannon, Chao, and ACE indices, was calculated by using QIIME (version 1.9.1). The principal coordinate analysis (PCoA) based on the Bray–Curtis dissimilarity matrix was conducted by using vegan (version 3.3.1). Linear discriminant analysis effect size (LEfSe) was determined, and the taxa with an LDA Score > 3 were considered as exhibiting a significant effect size. Spearmen’s rank correlation tested the relationships between the relative abundance (RA) of the ruminal bacteria (at genus level) and fermentation parameters (VFA concentration and MCP) using the “corrplot” package in R (version 3.4.1). Phylogenetic Investigation of Communities by Reconstruction of Unobserved States 2 (PICRUSt2) software predicted microbiota function and determined the differences among different treatment groups.

### 2.5 Statistical analysis of data

Data were analyzed by one- or two-way analysis of variance (ANOVA) for repeated measures data. Differences among treatment means were determined using the Duncan multiple comparison test. The *p*-value of <0.05 was taken to indicate statistical significance.

## 3 Results

### 3.1 Dynamic rumen protection rate of RU-Leu and RP-Leu *in situ* incubation

**Figure 1:**
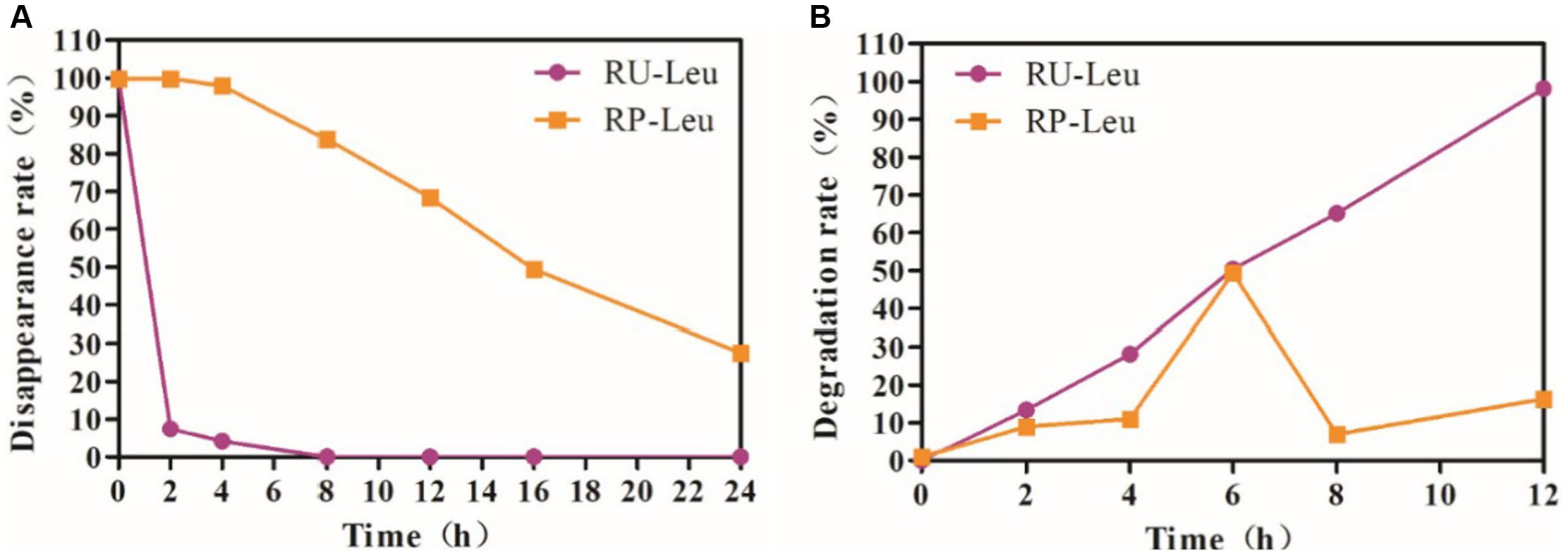
**(A)** The dynamic rumen protection rate of RU-Leu and RP-Leu *in situ*. **(B)** The dynamic rumen degradation rate of RU-Leu and RP-Leu *in vitro*. RU-Leu, rumen-unprotected *L*-leucine; RP-Leu, rumen-protected *L*-leucine.

As expected, *L*-leucine was completely degraded and disappeared (*p* < 0.01) at incubated 8 h compared with 0 h in the RU-Leu group, whereas only 16.20% in the RP-Leu group (*p* < 0.05; Figure 1A and Supplementary Table S1). The rumen disappearance was increased (*p* < 0.01) with incubated time increased and the rumen protection rate was 68.3% at incubated 12 h in the RP-Leu group.

### 3.2 Dynamic rumen degradation rate of RU-Leu and RP-Leu *in vitro* incubation

The rumen protection rate was greater (*p* < 0.01) in the RP-Leu group than in the RU-Leu group (Figure 1B and Supplementary Table S2). The RU-Leu product was completely degraded (*p* < 0.05) at incubated 12 h than at incubated 0 h, while the release rate of RP-Leu was the peak (*p* < 0.05) at incubated 6 h, there were no differences at other times in incubation progress (*p* > 0.05).

### 3.3 Fermentable substrate nutrient degradability and gas production

**Figure 2:**
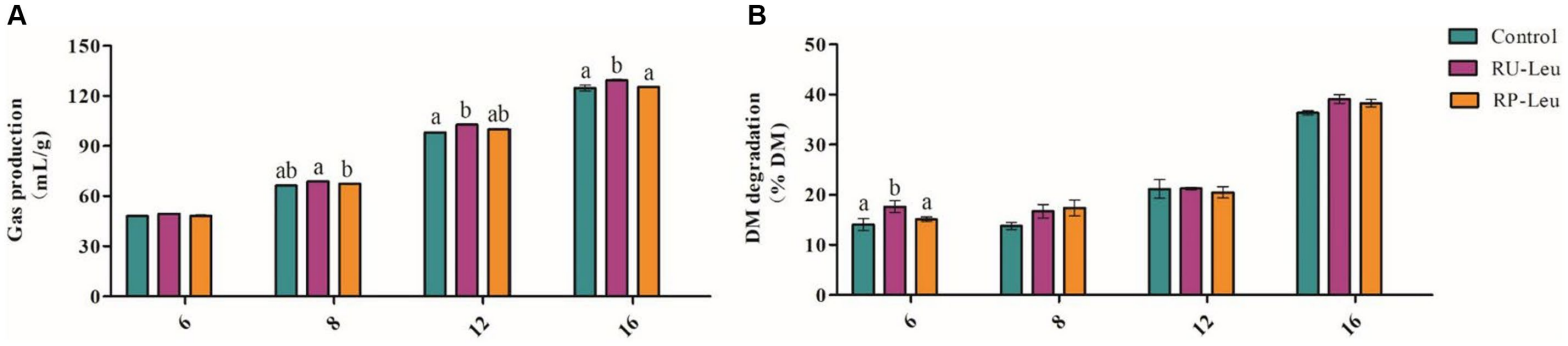
The effect of RU-Leu and RP-Leu on gas production **(A)** and DM degradability **(B)** of fermentable substrates. RU-Leu, rumen-unprotected *L*-leucine; RP-Leu, rumen-protected *L*-leucine; DM, dry matter. ^a,b^Different letters represent significantly different values (*p* < 0.05, *n* = 5).

The GP was greater (*p* < 0.05) in the RU-Leu group than in the RP-Leu group and control group at 8, 12, and 16 h and increased with incubated time increased (interaction, *p* < 0.001; Figure 2A and Supplementary Table S3). The dry matter degradability (DMD) (*p* < 0.1) (Figure 2B and Supplementary Table S3), neutral detergent fiber degradability (NDFD), and acid detergent fiber degradability (ADFD) were not affected (*p* > 0.05) by treatment and the interaction (*p* > 0.05) between treatment and incubated time, whereas increased (*p* < 0.05) with incubated time increased (Supplementary Table S3).

### 3.4 pH and fermentation parameters

The pH was greater (*p* < 0.05) in RU-Leu and RP-Leu groups than in the control group at 12 h, whereas no differences (*p* > 0.05) among 3 groups at 2, 4, 6, 8, and 16 h (interaction, *p* < 0.001; Table 2). Concentrations of total VFAs, propionate, butyrate, and branch-chained VFAs were increased (*p* < 0.001) in the RU-Leu group than in the control group and RP-Leu group, and interaction (*p* < 0.001). The concentration of NH_3_-N was greater (*p* < 0.05) in RU-Leu than in the control and RP-Leu groups at 4 and 12 h. The concentration of NH_3_-N was greater (*p* < 0.05) in the RU-Leu and RP-Leu group than in the control group at 16 h, whereas no differences (*p* > 0.05) between RU-Leu and RP-Leu group (interaction, *p* < 0.05). The concentration of MCP was greater (*p* < 0.05) in the RU-Leu group than control and RP-Leu group at 4 h (interaction, *p* < 0.05), and the RU-Leu group and RP-Leu group increased (*p* < 0.05) MCP concentration than the control group at 6 h.

**Table 2:** Rumen fermentation parameters (*n* = 6).

Items	Treatment^1^	Fermentation time (h)						SEM	*p*-value		
		2	4	6	8	12	16		Leu	Time	Leu × Time
pH	Control	6.90	6.89	6.87	6.80	6.75^a^	6.74	0.001	<0.01	<0.001	<0.001
	RU-Leu	6.93	6.89	6.90	6.82	6.80^b^	6.72				
	RP-Leu	6.94	6.91	6.88	6.84	6.80^b^	6.74				
**VFA, m*M***											
Total VFA	Control	31.8	36.2	41.8^a^	44.2^a^	56.60	63.90	0.180	0.04	< 0.001	0.285
	RU-Leu	32.5	37.3	42.6^b^	47.6^b^	54.50	65.70				
	RP-Leu	32.4	35.7	41.8^a^	46.6^a^	55.90	64.50				
Acetate(A)	Control	22.4	25.2	28.7	29.9	37.90	42.40	0.124	0.79	< 0.001	0.142
	RU-Leu	22.8	25.8	28.8	31.9	35.70	42.90				
	RP-Leu	22.7	24.8	28.7	31.6	37.50	42.60				
Propionate(P)	Control	6.02	7.02	8.36^a^	9.28	12.30	14.30	0.039	<0.01	<0.001	0.356
	RU-Leu	6.14	7.19	8.59^b^	9.84	11.80	14.30				
	RP-Leu	6.13	6.90	8.47^c^	9.80	12.10	14.60				
Butyrate	Control	2.75	3.23	3.78^a^	4.04	5.23	5.83	0.016	0.02	<0.001	0.252
	RU-Leu	2.79	3.33	3.85^b^	4.25	5.01	5.98				
	RP-Leu	2.80	3.20	3.80^a^	4.26	5.10	5.85				
Valerate	Control	0.19^a^	0.23	0.27^a^	0.29	0.38	0.47	0.094	<0.01	<0.001	0.255
	RU-Leu	0.20^ab^	0.24	0.29^b^	0.31	0.39	0.49				
	RP-Leu	0.21^b^	0.23	0.27^a^	0.30	0.38	0.46				
Iso-valerate	Control	0.25^a^	0.26^a^	0.28^a^	0.28^a^	0.39^a^	0.53^a^	0.375	<0.001	<0.001	<0.001
	RU-Leu	0.38^b^	0.47^b^	0.77^b^	0.96^b^	1.30^b^	1.55^b^				
	RP-Leu	0.26^a^	0.33^ab^	0.28^a^	0.32^c^	0.45^a^	0.56^a^				
Iso-butyrate	Control ^a^	0.22	0.23	0.26^a^	0.27	0.34	0.42	0.076	0.33	<0.001	<0.01
	RU-Leu ^b^	0.19	0.22	0.23^b^	0.28	0.35	0.44				
	RP-Leu ^a^	0.22	0.22	0.25^a^	0.28	0.34	0.42				
BCVFA	Control	0.68^a^	0.73	0.82^a^	0.85^a^	1.13^a^	1.41^a^	0.008	<0.001	<0.001	<0.001
	RU-Leu	0.79^b^	0.93	1.29^b^	1.54^b^	2.05^b^	2.49^b^				
	RP-Leu	0.69^a^	0.79	0.82^a^	0.92^a^	1.16^a^	1.45^a^				
A:P	Control	3.71	3.59	3.43	3.23	3.08	2.96	0.005	0.50	<0.001	0.354
	RU-Leu	3.72	3.58	3.36	3.24	3.02	3.01				
	RP-Leu	3.71	3.59	3.39	3.22	3.08	2.93				
NH_3_-N (mg/dL)	Control	6.66	5.05^a^	4.34^ab^	3.79	2.23^a^	4.37^a^	0.058	0.04	<0.001	<0.01
	RU-Leu	6.59	5.66^b^	4.76^a^	4.82	4.14^b^	5.99^b^				
	RP-Leu	6.70	5.79^b^	3.66^bc^	4.10	2.24^a^	4.85^ab^				
MCP (mg/dL)	Control	13.60	8.42^a^	12.10^a^	35.10	41.10	45.50	0.277	0.04	<0.001	0.025
	RU-Leu	15.70	12.2^b^	15.30^b^	42.10	41.60	47.30				
	RP-Leu	12.30	8.91^a^	15.30^b^	32.70	35.70	51.80				

One- and two-way analysis of variance (ANOVA) was used to compare the means. Values within the same column with different letters are significantly different (*p* < 0.05). VFA, volatile fatty acids; BCVFA, branched-chain VFA, including iso-butyrate, valerate, iso-valerate; A:P, acetate: propionate; NH_3_-N, ammonia -N; MCP, microbial crude protein. ^1^Control = basal TMR (no supplemental RP-Leu or RU-Leu); RU-Leu = basal TMR diet + 12 mg RU-Leu; RP-Leu = basal TMR die + 12 mg RP-Leu.

### 3.5 Free amino acids metabolism

**Figure 3:**
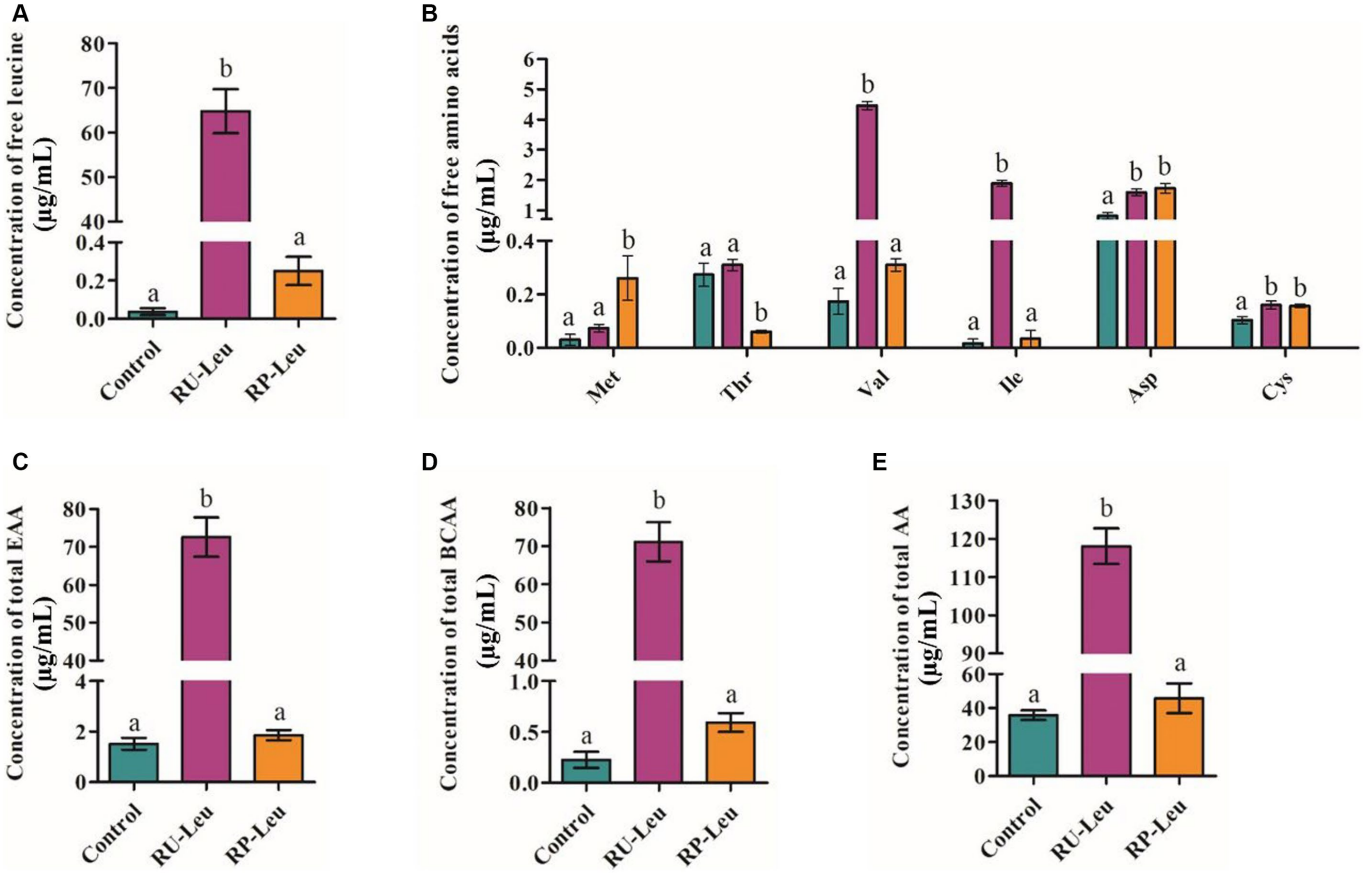
The effect of RU-Leu and RP-Leu on concentrations of free leucine amino acids **(A)**, free EAAs and NEAAs **(B)**, free total EAAs **(C)**, free total BCAAs **(D)**, and free total AAs **(E)** at 6 h *in vitro*. RU-Leu, rumen-unprotected *L*-leucine; RP-Leu, rumen-protected *L*-leucine; EAA, essential amino acids; BCAA, branched-chain amino acids. ^a,b^Different letters represent significantly different values (*p* < 0.05, *n* = 5).

Concentrations of asparagine and cysteine were greater (*p* < 0.05) in the RU-Leu and RP-Leu groups than in the control group at 6 h (Figure 3B and Supplementary Table S4). Concentrations of leucine, isoleucine, valine, total amino acids, essential amino acids, and total branch chain amino acids were greater (*p* < 0.001) in RU-Leu than in RP-Leu and control group (Figures 3A,C–E and Supplementary Table S4). The concentration of methionine was greater (*p* < 0.05), whereas threonine was lesser (*p* < 0.01) in the RP-Leu group than in the control group and RU-Leu group.

### 3.6 Microbial diversity and composition

To test whether leucine modulated rumen microbiota, we performed 16S rDNA gene sequencing to analyze the bacterial taxonomic composition in an incubation medium at 6 h. A total of 24 samples were obtained from three groups (*n* = 8) and subsequently sequenced to generate V1–V9 16S rRNA gene profiles. A total of 1,055,954,675 raw reads were generated from the incubation medium samples, and 706,400 high-quality sequences remained after quality filtering and removal of chimeric sequences. A total of 576 OTUs were obtained based on 97% nucleotide sequence identity analysis among reads, belonging to 145 species, 88 genera, 40 families, 27 orders, 14 classes, and 9 phyla.

**Figure 4:**
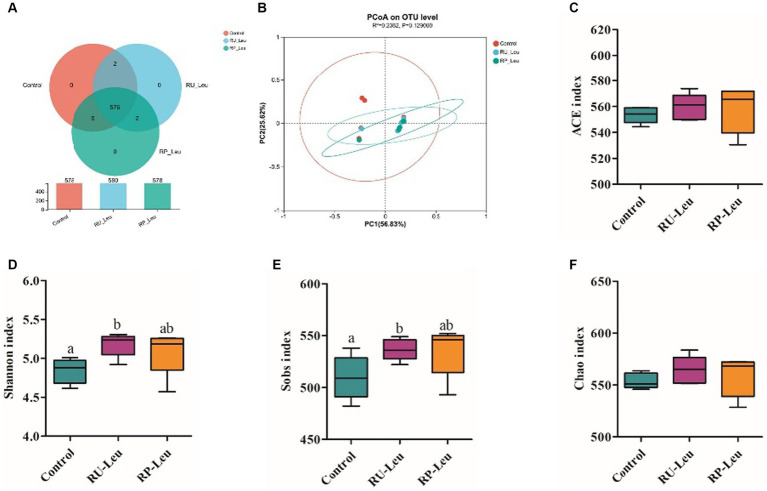
The effect of RU-Leu and RP-Leu on the composition of the microbiota. **(A)** OUT distribution across different treatment groups at 6 h *in vitro*. **(B)** PCoA score plot. **(C–F)** Alpha diversity evaluation of colon microbial richness and evenness by measuring Sobs, Shannon, ACE, and Chao diversity indexes. RU-Leu, rumen-unprotected *L*-leucine; RP-Leu, rumen-protected *L*-leucine. ^a,b^Different letters represent significantly different values (*p* < 0.05, *n* = 5).

**Figure 5:**
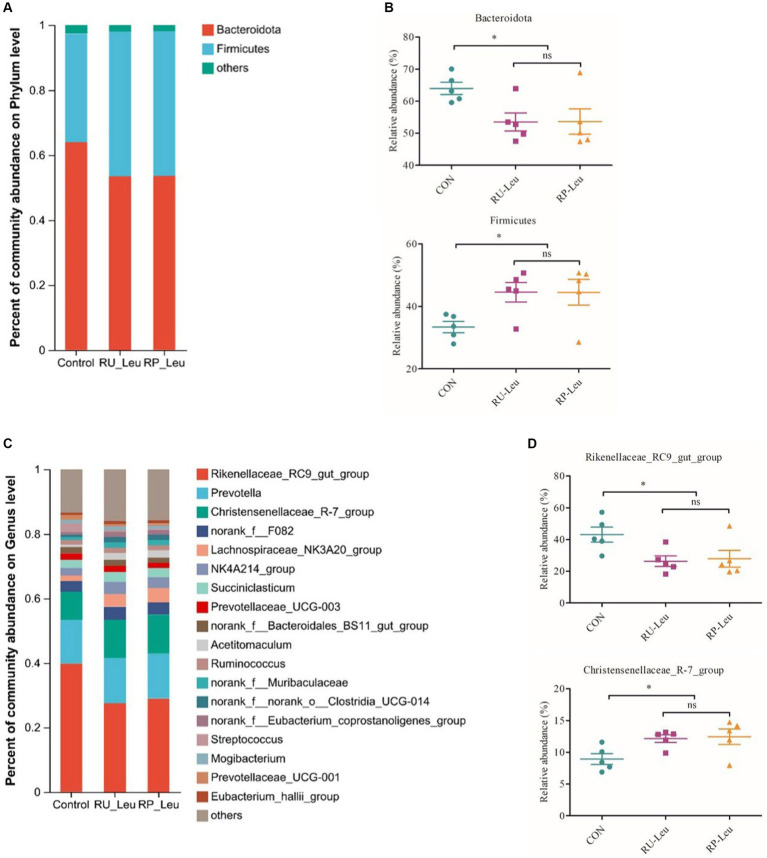
The effect of RU-Leu and RP-Leu on the composition of the microbiota at 6 h *in vitro*. **(A)** Relative abundance (%) of bacteria at the phylum level in the three groups (mean of each group). **(B)** The effect of Leu treatment on relative abundances of *Bacteroidota* and *Firmicutes*. **(C)** Relative abundances (%) of bacteria at the genus level in the three groups (mean of each group). **(D)** The effect of Leu treatment on relative abundances of *Rikenellaceae_RC9_gut_group* and *Christensenellaceae_R-7_group*. RU-Leu, rumen-unprotected *L*-leucine; RP-Leu, rumen-protected *L*-leucine. Data were shown as means (*n* = 5). Significant (*p* < 0.05) statistical differences were represented by (*).

The dilution curve tends to be gentle, and the coverage is close to 100%. Both of them indicate that the detection rate of the samples’ microbial community is close to saturation, and the current sequencing amount can cover most species in samples. A total of 576 OTUs were shared among the three treatment groups (Figure 4A). There was no difference in the number of specific OTUs among the three treatment groups. Alpha diversity reflects the richness and diversity of the microbiota. The Sobs and Shannon indices of alpha diversity of the incubation medium were greater (*p* < 0.05, *p* < 0.001) in the RU-Leu group than in the control group, there was no difference between RU-Leu and RP-Leu group (*p* > 0.05) (Figures 4D,E). There was no effect in the Chao and ACE indices among the treatment groups (*p* > 0.05) (Figures 4C,F). To measure the degree of similarity between microbial communities, *β*-diversity was further evaluated using Bray–Curtis PCoA. The PCoA revealed not affected by different treatment groups (*p* > 0.05) (Figure 4B). At the phylum level, *Bacteroidotas* and *Firmicutes* were predominant in both groups (Figures 5A,B and Supplementary Table S5). The RU-Leu group and RP-Leu group decreased (*p* < 0.05) the relative abundance (RA) of *Bacteroidotas*, and increased (*p* < 0.05) the RA of *Firmicutes* compared to the control group (Supplementary Table S5). At the genus level, *Rikenellaceae_RC9_gut_group* and *Christensenellaceae_R-7_group* were predominant in both groups (Figures 5C,D and Supplementary Table S6). The RU-Leu group and RP-Leu group decreased (*p* < 0.05) the RA of *Rikenellaceae_RC9_gut_group*, *Prevotellaceae_UCG-001*, *norank_f__norank_o__WCHB1-41*, increased (*p* < 0.05) the RA of *Christensenellaceae_R-7_group*, *Lachnospiraceae_NK3A20_group*, *NK4A214_group*, *Acetitomaculum*, *norank_f__Muribaculaceae*, *norank_f__norank_o__Clostridia_UCG-014*, *norank_f__norank_o__WCHB1-41*, *U29-B03*, and *p-1088-a5_gut_group* compared to the control group (Supplementary Table S6).

**Figure 6:**
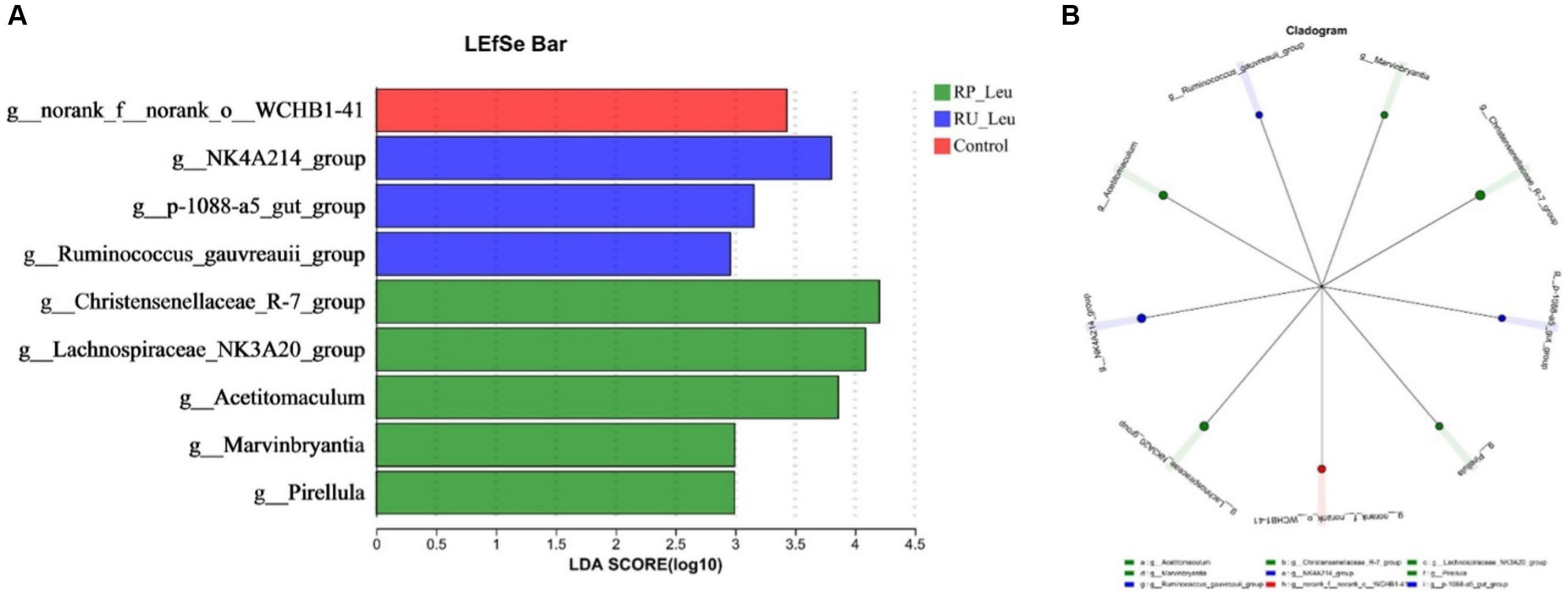
**(A)** Linear discriminant analysis effect size (LEfSe) was performed to identify the bacteria that are differentially represented between the different groups. **(B)** Cladogram reported. RU-Leu, rumen-unprotected *L*-leucine; RP-Leu, rumen-protected *L*-leucine. Data were shown as means (*n* = 5). Significant (*p* < 0.05) statistical differences were represented by (*).

Differential microbiota that varied with leucine treatments were further identified using linear discriminant analysis effect size (LEfSe Bar) and Cladogram (Figures 6A,B). With a default LDA cutoff ±2.5, differential taxa totaling 1, 3, and 5 in control, RU-Leu, and RP-Leu groups, respectively. The bacteria biomarkers in the control group were *g_norank_f_norank_o_WCHB1-41*, and in the RU-Leu group were *g_NK4A214*_*group*, *g_p-1088-a5_gut_group*, and *g_Ruminococcus_gauvreauii_group*, and in the RP-Leu group were *g_Christensenellaceae_R-7_group*, *g_Lachnospiraceae_NK3A20_group*, *g_Acetitomaculum*, *g_Marvinbryantia*, and *g_Pirellula*.

### 3.7 Correlations between ruminal microbiota and fermentation parameters

**Figure 7:**
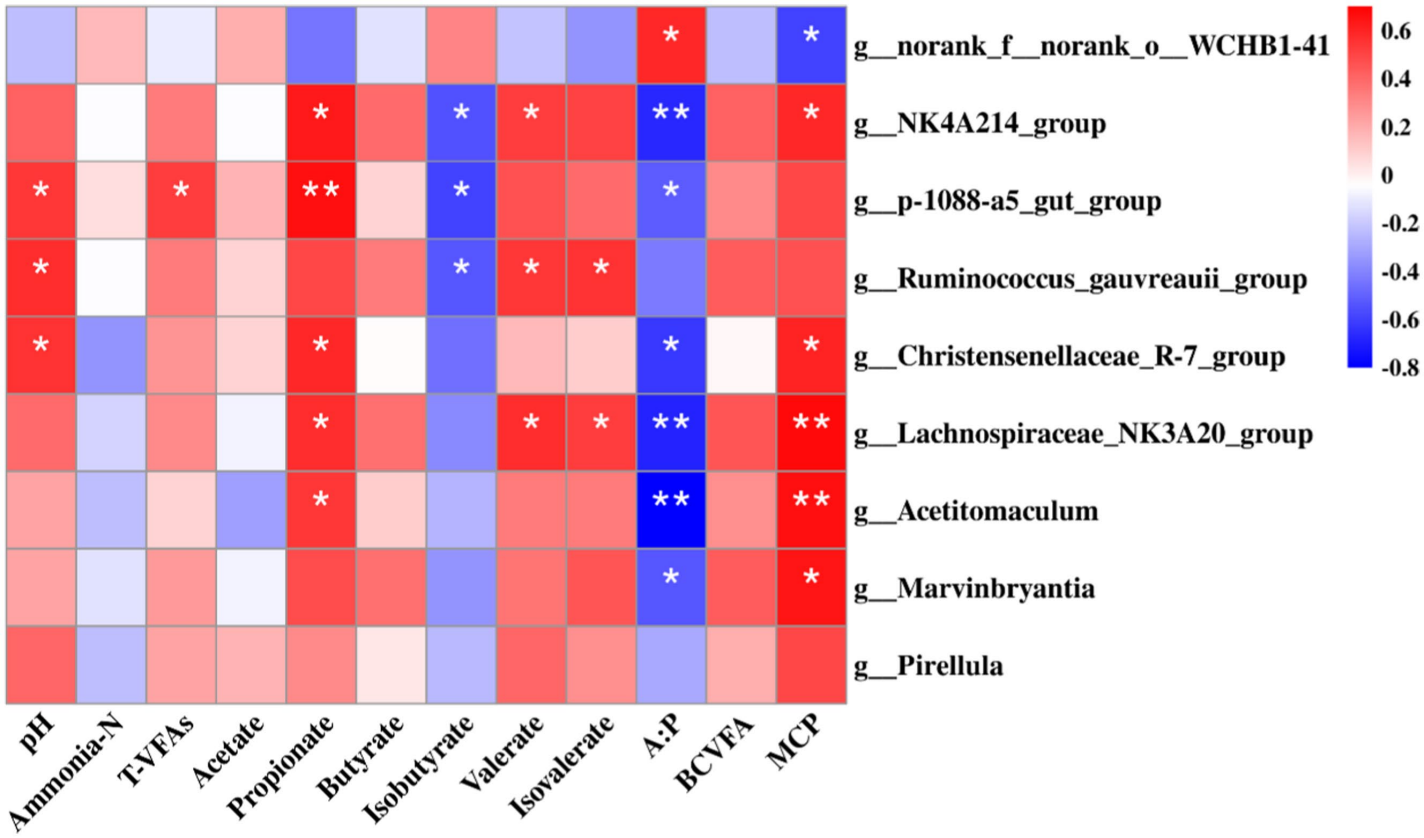
Spearman’s rank correlation analysis between bacteria (76.65% of the total bacterial relative abundance) at the genus level and rumen fermentation parameters, including pH, microbial protein (MCP), ammonia-N, T-VFAs (total volatile fatty acids), acetate, propionate, butyrate, isobutyrate, valerate, isovalerate, acetate: propionate (A:P), and branched chain volatile fatty acids (BCVFA). RU-Leu, rumen-unprotected *L*-leucine; RP-Leu, rumen-protected *L*-leucine. Data were shown as means (*n* = 5). Significant (*p* < 0.05) or (*p* < 0.01) statistical differences were represented by (*) or (**).

A Spearman rank correlation tested the relationships between ruminal microbiota (genus level) and fermentation parameters. A total of 20 positive (*p* < 0.05) and 10 negative (*p* < 0.05) correlations emerged (Figure 7). The concentration of MCP was correlated positively with *Acetitomaculum* and *Lachnospiraceae_NK3A20_group* (*p* < 0.01), *Christensenellaceae_R-7_group*, *Marvinbryantia*, and *NK4A214_group* (*p* < 0.05), and negatively with *norank_f_norank_o_WCHB1-41* (*p* < 0.05). The proportion of propionate were correlated positively with *p-1088-a5_gut_group* (*p* < 0.01), *NK4A214_group*, *Lachnospiraceae_NK3A20_group*, *Christensenellaceae_R-7_group*, and *Acetitomaculum* (*p* < 0.05). The proportion of valerate and isovalerate were correlated positively with *NK4A214_group*, *Lachnospiraceae_NK3A20_group*, and *Ruminococcus_gauvreauii_group* (*p* < 0.05). The proportion of isobutyrate was correlated negatively with *NK4A214_group*, *p-1088-a5_gut_group*, and *Ruminococcus*_*gauvreauii_group* (*p* < 0.05). The ratio of A:P was correlated positively with *norank_f_norank_o_WCHB1-41* (*p* < 0.05), and negatively with *Acetitomaculum*, *Lachnospiraceae_NK3A20_group*, and *NK4A214_group* (*p* < 0.01), *Christensenellaceae_R-7_group*, *Marvinbryantia*, and *p-1088-a5_gut_group* (*p* < 0.05). The pH was correlated positively with *Christensenellaceae_R-7_group*, *p-1088-a5_gut_group*, and *Ruminococcus_gauvreauii_group* (*p* < 0.05).

### 3.8 PICRUSt2 function prediction

**Figure 8:**
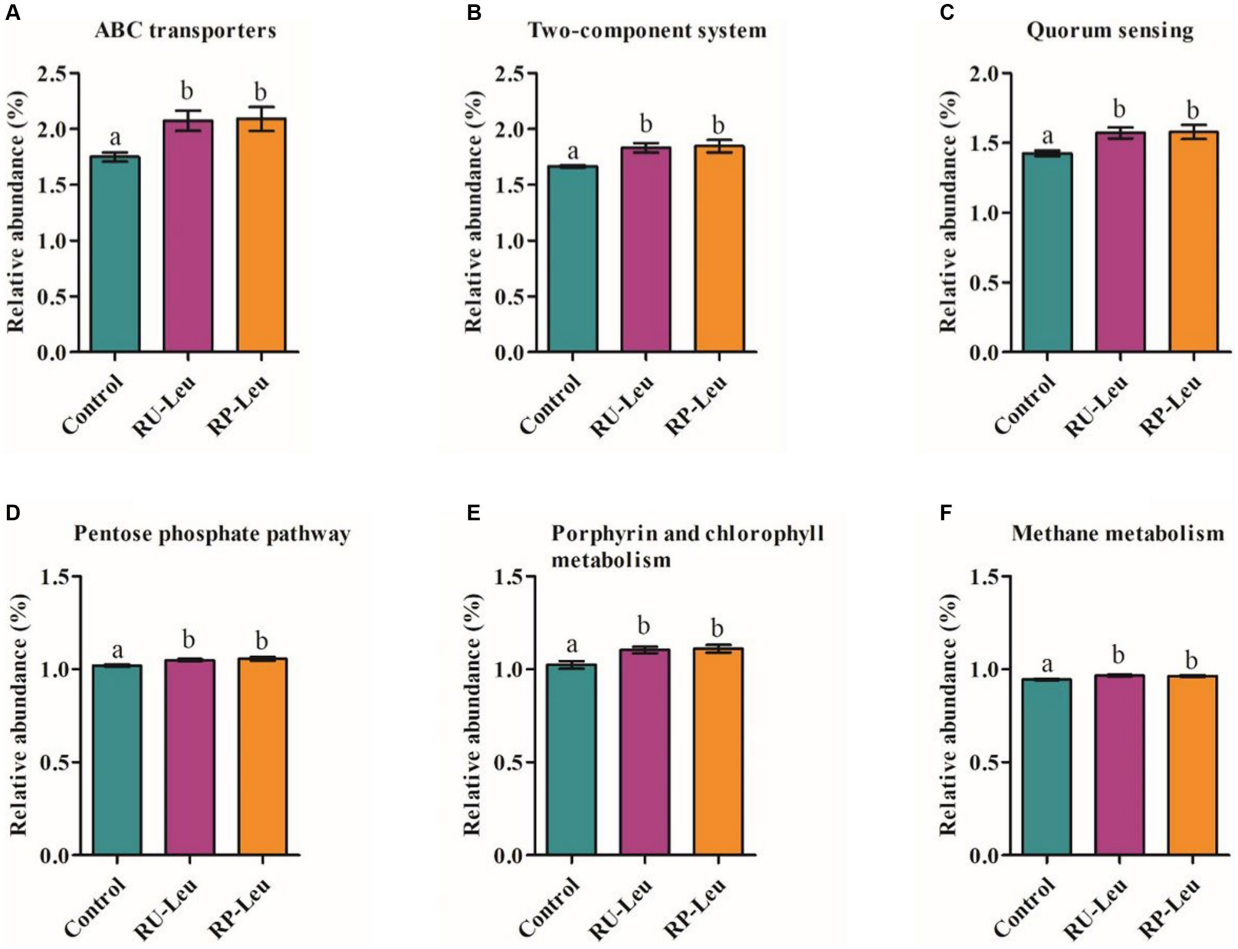
**(A–F)** Significant differences in the functional pathways among different treatment groups. RU-Leu, rumen-unprotected *L*-leucine; RP-Leu, rumen-protected *L*-leucine. ^a,b^Different letters represent significantly different values (*p* < 0.05, *n* = 5).

The top 40 functions of the rumen bacterial communities in the control and leucine treatment groups were predicted. Picrust2 identified 6 predictive metabolism pathways that were affected (*p* < 0.05) by the supplement leucine (Supplementary Table S7). Overall, the most abundant pathway was Metabolic pathways (25.57%), followed by Biosynthesis of secondary metabolites(13.15%). ABC transporters, Two-component system, Quorum sensing, Porphyrin and chlorophyll metabolism, Pentose phosphate pathway, and Methane metabolism were greater (*p* < 0.05) in the RU-Leu group and RP-Leu group than the control group (Figures 8A–F).

## 4 Discussion

Clarifying the degradation rate of amino acids in the rumen is beneficial for determining their application forms and improving their bioavailability (Gilbreath et al., 2019, 2020). The degrading of dietary AAs by ruminal bacteria has long been understood (Tan et al., 2021). However, to date, how much AAs are degraded by ruminal bacteria and the degradation rate of all AAs remains inconsistent (Gilbreath et al., 2020; Yanibada et al., 2020). Results in *in situ* experiments showed that the RP-Leu was more stable in the rumen than RU-Leu, and effectively escaped degradation by rumen microbial, this result was consistent with rumen-protected limit AAs (lysine and methionine) in growing Tan lamps (Liu et al., 2021) studies. Additionally, the RU-Leu was slowly degraded compared to other amino acids. Based on the preliminary *in situ* incubation results, we suggested that the RP-Leu conforms to the characteristics of rumen protection, and be applied in *in vitro* fermentation. Results from the *in vitro* incubation experiments were again consistent with *in situ*, indicating that the RP-Leu product had a better protective ability in the rumen. Meanwhile, we found that free leucine was released rapidly from the RP-Leu and peaked at 6 h, but the concentration of free leucine was much less than the RU-Leu. Generally, the maximum ruminal retention time was usually 12–24 h in the case of powdery amino acids (Suh et al., 2022). Above present results, the RU-Leu product was completely degraded at least 8 h, indicating that *L-*leucine was more slowly than essential amino acids, the result was similar to methionine studies (Onodera, 1993).

Gas production reflects the extent of ruminal microbiota fermentation and it is highly correlated to dry matter digestibility (Saleem et al., 2019). In the present study, the Leu treatment groups increased the GP, but the dry matter digestibility of fermentable substrates was increased in the RU-Leu group only at 6 h and did not affect the digestibility of neutral detergent fiber and acid detergent fiber. The reason may be related to the rumen degrades free leucine that is not protected from attack, resulting in ammonia, CO_2_, VFAs, and branched-chain fatty acids (Bergen, 1979; Judd and Kohn, 2018; Tan et al., 2021). The ruminal pH, which is influenced by the concentrations of VFAs and ammonia-N, affects the growth and proliferation of ruminal microbes (Liu et al., 2022). In *in vitro* incubation results showed that the incubation fluid pH was within the optimal values of 6.2–7.2 (Hultquist and Casper, 2016), indicating that fermentation progress was normal. VFAs are the primary products of ruminal microbiota fermentation, VFA concentrations may reflect changes in rumen fermentation patterns (Reis et al., 2016; Beckett et al., 2021). In the present study, we found that free leucine, particularly from RU-Leu, which increased the concentrations of branched-chain VFAs (isobutyrate, valerate, and isovalerate) and propionate at 6 h, did not affect acetate and acetate to propionate ratio, whereas the RP-Leu degraded partly. These results showed that free leucine could change the fermentation patterns to increase the propionate and branched-chain VFAs molar proportion, the results were consistent with the previous study (Yang, 2002; Zhang et al., 2013; Liu et al., 2021). Besides, it may be associated with the coating material in RP-Leu, the palm oil regulated the rumen fermentation trend to propionate pattern by changing the metabolism of fatty acids (Jal et al., 2009; Beckett et al., 2021). Previous studies (Allison and Bryant, 1963; Apajalahti et al., 2019) have shown that branched-chain VFAs play an important role in improving apparent dry matter digestibility, and microbial growth, and enhancing microbial functions, and enzyme activities. Moreover, the free leucine from RU-Leu improved the proportion of BCAAs converted to BCVFAs, thus, the increase in BCVFAs concentration may indicate a greater availability of BCAAs *in vitro* rumen (Schulmeister et al., 2019). Microbial crude proteins (MCP) would then be available for digestion in the abomasum and the small intestine to release BCAAs (Gilbreath et al., 2020), while ammonia-N concentrations were closely related to rumen microbial crude proteins synthesis (Hristov and Broderick, 1994), which optimal ranged from 3 to 8 mg/dL with *in vitro* fermentation (Clark et al., 1992; Yáñez-Ruiz et al., 2016). We found that the NH_3_-N concentration was within the optimal range, and the RP-Leu and RU-Leu increased MCP synthesis at 6 h in the present study. This occurred because BCVFAs as the primary products of leucine catabolism, which promotes the growth of ruminal bacteria to the utilization of NH_3_-N, resulting in MCP synthesis increased (Allison, 1969; Cummins and Papas, 1985; Zhang et al., 2013).

Combined with all findings, we suggested that fermentation parameters changed may be closely related to the amino acid metabolism and bacteria composition when incubated at 6 h. Previous have reported that the amino acids in ruminal fluid have a stimulatory effect on the growth of ruminal bacteria, even in the case of abundant NH_3_ and carbohydrates (Cotta and Russell, 1982; Argyle and Baldwin, 1989). In the present study, the results show that the RP-Leu and RU-Leu groups increased the concentrations of asparagine, glutamine, and cysteine, whereas free leucine from the RU-Leu increased total amino acids, essential amino acids, and BCAAs concentrations. These findings were consistent with previous studies’ *in vitro* fermentation for BCAAs (Zhang et al., 2013), and for EAAs (Hassan et al., 2021). Therefore, we think that free leucine could alter the amino acids metabolism and composition, promote the BCAAs conversion to BCVFAs, and then change the fermentation parameters (Allison et al., 1966).

Ruminal microorganisms play important roles in rumen development, nutrient digestion, and host physiological function, and dominant phyla *Bacteroidetes* to *Firmicutes* ratio (F: B) is beneficial for animal growth (Xin et al., 2019; Huang et al., 2021). In the present study, we found that free leucine from the RU-Leu group increased the Sobs and Shannon indices of alpha diversity, but no difference between the RU-Leu and RP-Leu groups. In addition, free leucine from the RU-Leu and RP-Leu groups decreased the relative abundance (RA) of *Bacteroidetes*, and of *Firmicutes* increased, and increased the F: B ratio, which is in line with earlier study *in vitro* fermentation (Hassan et al., 2021) and in Tan lamps (Liu et al., 2022), the reason of change in dominant phyla may be associated with free leucine in rumen fluid stimulated the grow and proliferation of *Firmicutes* bacterial by product BCVFAs. At the genu level, *Rikenellaceae_RC9_gut_group*, a member of the *Rikenellaceae* family, degrades cellulose and hemicellulose to produce acetate (Liu et al., 2022), however, the RA of *Rikenellaceae_RC9_gut_group* decreased in the RU-Leu group and RP-Leu group compared with the control group, indicating that free leucine may alter the rumen fermentation patterns to increase the propionate production, these results were consistent with a study in cattle (Xin et al., 2019). Moreover, free leucine from the RU-Leu and RP-Leu groups increased the RA of *Christensenellaceae_R-7_group* bacteria. The *Christensenellaceae_R-7_group*, belonging to the family *Christensenellaceae*, produces acetate and butyrate, with the ability to utilize arabinose, glucose, and mannose (Liu et al., 2022), indicating that free leucine could promote the utilization of carbohydrates for ruminants. Therefore, changes in the relative abundance of ruminal microorganisms may be a critical mechanism leading to rumen fermentation patterns.

According to the results of variance analysis and Spearman correlation analysis, we found that *p-1088-a5_gut_group*, *NK4A214_group*, *Lachnospiraceae_NK3A20_group*, *Christensenellaceae_R-7_group*, and *Acetitomaculum* were positively correlated with propionate, which is in agreement with previous studies (Beckett et al., 2021; Liu et al., 2022). Additionally, *Acetitomaculum*, *Lachnospiraceae_NK3A20_group*, *Christensenellaceae_R-7_group*, *Marvinbryantia*, and *NK4A214_group* were positively correlated with MCP, which is by the reports that the bacterial are cellulolytic (Liu et al., 2022). Free leucine from the RU-Leu and RP-Leu increased the relative abundance of these bacteria, and this is beneficial to rumen development and the metabolizable microbial crude protein synthesis for ruminants (Firkins et al., 2007). Branched-chain VFA from the fermentation of branched-chain AA, which is either used for amino acids resynthesis, or as a growth factor for ruminal bacteria (Allison et al., 1958). In our study, although there are no bacteria positively or negatively correlated with BCVFA, we infer that an increase in its concentration could stimulate the growth of bacteria of MCP synthesis and propionate product.

By using PICRUSt2 to predict the potential functions of bacteria, numerous metabolism-related pathways emerged. The most prominent functional categories were ABC transporters at the KEGG level 3 metabolic categories. ABC transporters are vital for the survival and productivity of ruminal bacteria (Higgins, 2001). In the present study, 6 predictive metabolic pathways were affected by RU-Leu and RP-Leu. The BCAAs are vital contributors to MCP synthesis and BCVFA product (Allison, 1969; Firkins et al., 2007), the fact that free leucine from RU-Leu and RP-Leu increased MCP and BCVFA concentration in the present study.

## 5 Conclusion

Confirmed that the rumen protection rate of rumen-protected Leu was greater than the rumen-unprotected Leu. Extra free leucine released by the rumen-protected or unprotected Leu, which altered ruminal bacterial composition with decreased *Bacteroidetes* to *Firmicutes* ratio, changed fermentation parameters with increased branched-chain VFAs and microbial crude protein production, shifted fermentation pattern by facilitating propionate production, and regulated amino acid metabolism with promoted conversion between branched-chain AA and branched chain VFAs. In terms of microbial crude protein synthesis and propionate production, the RU-Leu was more effective than the RP-Leu, whereas the RP-Leu was more stable in the rumen. Therefore, the RP-Leu was suitable for ruminant production applications in the future.

## Data availability statement

The datasets presented in this study can be found in online repositories. The names of the repository/repositories and accession number(s) can be found below: NCBI – PRJNA1013731.

## Ethics statement

The animal study was approved by the Ethics Committee on Animal Use of the Hunan Agricultural University. The study was conducted in accordance with the local legislation and institutional requirements.

## Author contributions

JA: Data curation, Investigation, Writing – original draft. WS: Formal analysis, Funding acquisition, Methodology, Supervision, Writing – review & editing. HL: Writing – review & editing. CY: Writing – review & editing. KC: Writing – review & editing. QY: Writing – review & editing. ZL: Writing – review & editing. DX: Funding acquisition, Supervision, Writing – review & editing. ZW: Writing – review & editing. XL: Writing – review & editing. LL: Formal analysis, Funding acquisition, Methodology, Supervision, Writing – review & editing. FW: Conceptualization, Funding acquisition, Methodology, Project administration, Supervision, Writing – review & editing.

## Footnotes

^1^ https://cloud.majorbio.com
