# Supplementary material for: Comparison of the effects of rumen-protected and unprotected L-leucine on fermentation parameters, bacterial composition, and amino acids metabolism in in vitro rumen batch cultures
Source: Front Microbiol. 2023 Nov 23;14:1282767. doi: 10.3389/fmicb.2023.1282767 (PMC10701908; doi:10.3389/fmicb.2023.1282767)
Supplement: Supplementary file 1 [file Data_Sheet_1.docx]

**Appendix: Supplementary Tables**

**Table S1**

The dynamic rumen protection rate of RU-Leu and RP-Leu *in situ* (n = 8).

| Time (h) | Residue（%） | |
| --- | --- | --- |
|  | RU-Leu | RP-Leu |
| 0 | 99.90^a^ | 99.90^a^ |
| 2 | 7.24^b^ | 99.80^a^ |
| 4 | 4.14^b^ | 97.90^a^ |
| 8 | 0 | 83.80^b^ |
| 12 | 0 | 68.30^b^ |
| 16 | 0 | 49.40^b^ |
| 24 | 0 | 27.60^b^ |
| SEM | 14.14 | 5.12 |
| *P*-value | < 0.001 | < 0.001 |

One-way analysis of variance (ANOVA) was used to compare the means. Values within the same column with different letters are significantly different (*P* < 0.05).

**Table S2**

The dynamic degradation rate of RU-Leu and RP-Leu *in vitro* (n = 6).

| Time (h) | Degradation（%）^1^ | |
| --- | --- | --- |
|  | RU-Leu | RP-Leu |
| 2 | -13.40 | +10.00 |
| 4 | -45.20 | +13.00 |
| 6 | -62.20 | +51.00 |
| 8 | -73.50 | +28.00 |
| 12 | -98.50 | +25.00 |

“-” = degradation rate, “+” = release rate;

^1^ Calculation formula: C _free leucine content_ = C _incubation medium + leucine_ – C _incubation medium_;

Leu degradation rate % =（C_0_-C_X_）/C_0_ × 100;

C_0_: content of 0 h fermentation; C_X_: content of *X* h fermentation.

**Table S3**

Gas production and nutrition degradability *in vitro* (n = 6).

| Items | Treatments^1^ | Fermentation Time (h) | | | | SEM | *P*-value | | |
| --- | --- | --- | --- | --- | --- | --- | --- | --- | --- |
|  |  | 6 | 8 | 12 | 16 |  | Leu | Time | Leu×Time |
| GP  (mL/g) | Control | 49.20 | 66.60^ab^ | 111.0^a^ | 122.0^a^ | 0.11 | < 0.01 | < 0.001 | < 0.001 |
|  | RU-Leu | 50.20 | 68.80^a^ | 118.0^b^ | 130.0^b^ |  |  |  |  |
|  | RP-Leu | 48.40 | 66.10^b^ | 113.0^ab^ | 126.0^a^ |  |  |  |  |
| DMD  (% DM) | Control | 14.10^a^ | 14.80 | 21.20 | 36.30 | 0.23 | 0.05 | < 0.001 | 0.41 |
|  | RU-Leu | 17.60^b^ | 16.70 | 21.30 | 39.10 |  |  |  |  |
|  | RP-Leu | 15.10^a^ | 17.40 | 20.50 | 38.20 |  |  |  |  |
| NDFD  (% DM) | Control | 3.64 | 3.32 | 6.54 | 23.50 | 0.36 | 0.63 | < 0.001 | 0.41 |
|  | RU-Leu | 2.07 | 3.43 | 4.88 | 27.20 |  |  |  |  |
|  | RP-Leu | 3.20 | 3.32 | 6.52 | 27.30 |  |  |  |  |
| ADFD  (% DM) | Control | 2.56 | 3.10 | 3.36 | 19.20 | 0.52 | 0.11 | < 0.001 | 0.42 |
|  | RU-Leu | 2.97 | 3.17 | 3.51 | 23.20 |  |  |  |  |
|  | RP-Leu | 3.20 | 3.28 | 3.87 | 24.10 |  |  |  |  |

One- and two- way analysis of variance (ANOVA) was used to compare the means. Values within the same column with different letters are significantly different (*P* < 0.05).

^1^ Control = basal TMR ( no supplemental RP-Leu or RU-Leu); RU-Leu = basal TMR +12 mg RU-Leu; RP-Leu = basal TMR +16 mg RP-Leu. GP = gas production; DMD = dry matter degradability; NDFD = neutral detergent fiber degradability; ADFD = acid detergent fiber degradability.

**Table S4**

Concentration of free amino acids in incubation medium at 6 h *in vitro* (n = 4, µg/mL).

| Amino acids | Control | RU-Leu | RP-Leu | SEM | *P*-value |
| --- | --- | --- | --- | --- | --- |
| Met | 0.03^a^ | 0.07^a^ | 0.26^b^ | 0.04 | 0.03 |
| Thr | 0.27^a^ | 0.31^a^ | 0.06^b^ | 0.04 | < 0.01 |
| Val | 0.17^a^ | 4.45^b^ | 0.31^a^ | 0.70 | < 0.001 |
| Ile | 0.02^a^ | 1.88^b^ | 0.03^a^ | 0.31 | < 0.001 |
| Leu | 0.03^a^ | 64.76^b^ | 0.25^a^ | 10.90 | < 0.001 |
| Phe | 0.02 | 0.09 | 0.02 | 0.01 | 0.23 |
| Lys | 0.96 | 0.96 | 0.92 | 0.04 | 0.91 |
| Total EAA | 1.50^a^ | 72.50^b^ | 1.80^a^ | 11.90 | < 0.001 |
| Asp | 0.81^a^ | 1.59^b^ | 1.72^b^ | 0.16 | < 0.01 |
| Cys | 0.10^a^ | 0.16^b^ | 0.15^b^ | 0.01 | 0.03 |
| Ser | 0.17 | 0.16 | 0.15 | 0.01 | 0.77 |
| Glu | 4.85 | 7.88 | 6.82 | 0.60 | 0.13 |
| Gly | 0.54 | 0.55 | 0.50 | 0.02 | 0.75 |
| Ala | 1.03 | 1.08 | 0.97 | 0.09 | 0.92 |
| Tyr | 0.03 | 0.03 | 0.01 | 0.01 | 0.62 |
| His | 0.39 | 0.37 | 0.36 | 0.02 | 0.93 |
| Arg | 0.49 | 0.69 | 0.65 | 0.04 | 0.25 |
| Pro | 0.47 | 0.93 | 0.57 | 0.11 | 0.26 |
| Total NEAA | 8.91 | 13.40 | 11.90 | 0.97 | 0.15 |
| Total BCAA^1^ | 0.22^a^ | 71.09^b^ | 0.59^a^ | 9.98 | < 0.001 |
| Total AA | 35.70^a^ | 118.00^b^ | 45.80^a^ | 13.30 | < 0.001 |

One-analysis of variance (ANOVA) was used to compare the means. Values within the same row with different letters are significantly different (*P* < 0.05).

Control = basal TMR ( no supplemental RP-Leu or RU-Leu); RU-Leu = basal TMR +12 mg RU-Leu; RP-Leu = basal TMR +16 mg RP-Leu. Total EAA = total essential amino acids; Total NEAA = total non-essential amino acids; Total BCAA = total branched-chain amino acids; Total AA = total amino acids;

^1^ Total BCAA : include leucine, isoleucine, and valine.

**Table S5**

Relative abundance (%) of bacteria at the phylum level in the 3 groups at 6 h *in vitro* (n = 5).

| Items | Control | RU-Leu | RP-Leu | SEM | *P*-value |
| --- | --- | --- | --- | --- | --- |
| *Bacteroidota* | 63.99^a^ | 53.51^b^ | 53.66^b^ | 2.08 | 0.04 |
| *Firmicutes* | 33.35^a^ | 44.53^b^ | 44.52^b^ | 2.19 | 0.04 |
| *Verrucomicrobiota* | 0.98^a^ | 0.43^b^ | 0.31^b^ | 0.11 | 0.02 |
| *Planctomycetota* | 0.51 | 0.57 | 0.61 | 0.03 | 0.45 |
| *Proteobacteria* | 0.57 | 0.25 | 0.17 | 0.09 | 0.21 |
| *Actinobacteriota* | 0.13^a^ | 0.34^b^ | 0.37^b^ | 0.04 | 0.02 |
| *Desulfobacterota* | 0.25 | 0.21 | 0.24 | 0.03 | 0.89 |
| *Spirochaetota* | 0.10 | 0.05 | 0.07 | 0.01 | 0.42 |
| *unclassified_k__norank_d__Bacteria* | 0.04 | 0.03 | 0.02 | 0.006 | 0.32 |
| *Chloroflexi* | 0.02 | 0.04 | 0.02 | 0.005 | 0.30 |
| *WPS-2* | 0.04 | 0.02 | 0.01 | 0.006 | 0.05 |

One-analysis of variance (ANOVA) was used to compare the means. Values within the same row with different letters are significantly different (*P* < 0.05).

Control = basal TMR ( no supplemental RP-Leu or RU-Leu); RU-Leu = basal TMR +12 mg RU-Leu; RP-Leu = basal TMR +16 mg RP-Leu.

**Table S6**

Relative abundance (%) of bacteria at the genus level in the 3 groups at 6 h *in vitro* (n = 5).

| Items | Control | RU-Leu | RP-Leu | SEM | *P*-value |
| --- | --- | --- | --- | --- | --- |
| *Rikenellaceae_RC9_gut_group* | 41.08^a^ | 28.32^b^ | 29.90^b^ | 3.70 | 0.03 |
| *Prevotella* | 14.02 | 14.52 | 14.36 | 1.87 | 0.99 |
| *Christensenellaceae_R-7_group* | 8.93^a^ | 12.13^b^ | 12.45^b^ | 0.65 | 0.04 |
| *norank_f__F082* | 3.42 | 4.31 | 3.90 | 0.203 | 0.21 |
| *Lachnospiraceae_NK3A20_group* | 1.78^a^ | 4.01^b^ | 4.54^b^ | 0.41 | < 0.01 |
| *NK4A214_group* | 2.48^a^ | 3.88^b^ | 3.57^b^ | 0.23 | 0.02 |
| *Succiniclasticum* | 2.50 | 3.16 | 2.93 | 0.16 | 0.26 |
| *Prevotellaceae_UCG-003* | 2.09 | 1.99 | 1.66 | 0.16 | 0.56 |
| *norank_f__Bacteroidales_BS11_gut_group* | 1.99 | 1.91 | 1.59 | 0.12 | 0.43 |
| *Acetitomaculum* | 0.86^a^ | 2.17^b^ | 2.38^b^ | 0.24 | 0.01 |
| *Ruminococcus* | 1.46 | 1.74 | 1.65 | 0.11 | 0.61 |
| *norank_f__Muribaculaceae* | 0.89^a^ | 1.66^b^ | 1.58^b^ | 0.14 | 0.04 |
| *norank_f__norank_o__Clostridia_UCG-014* | 0.69^a^ | 1.63^b^ | 1.75^b^ | 0.20 | 0.04 |
| *norank_f__Eubacterium_coprostanoligenes_group* | 0.94 | 1.64 | 1.45 | 0.12 | 0.05 |
| *Streptococcus* | 2.72 | 0.70 | 0.38 | 0.63 | 0.28 |
| *Mogibacterium* | 1.11 | 1.08 | 1.01 | 0.06 | 0.85 |
| *Prevotellaceae_UCG-001* | 1.55^a^ | 0.67^b^ | 0.66^b^ | 1.66 | 0.02 |
| *UCG-004* | 0.83 | 0.99 | 0.99 | 0.071 | 0.59 |
| *Eubacterium_hallii_group* | 0.69 | 1.04 | 0.99 | 0.086 | 0.20 |
| *norank_f__UCG-010* | 0.72 | 0.96 | 0.98 | 0.071 | 0.27 |
| *Papillibacter* | 0.64 | 0.78 | 0.81 | 0.041 | 0.19 |
| *norank_f__norank_o__RF39* | 0.33 | 0.75 | 0.79 | 0.094 | 0.07 |
| *Butyrivibrio* | 0.47 | 0.62 | 0.60 | 0.057 | 0.55 |
| *Veillonellaceae_UCG-001* | 0.54 | 0.56 | 0.52 | 0.062 | 0.98 |
| *UCG-002* | 0.55 | 0.50 | 0.45 | 0.061 | 0.80 |
| *norank_f__Bacteroidales_RF16_group* | 0.23 | 0.65 | 0.55 | 0.084 | 0.09 |
| *norank_f__norank_o__WCHB1-41* | 0.79^a^ | 0.34^b^ | 0.25^b^ | 0.090 | 0.01 |
| *Lachnospiraceae_AC2044_group* | 0.24 | 0.59 | 0.54 | 0.069 | 0.07 |
| *Saccharofermentans* | 0.24 | 0.50 | 0.53 | 0.062 | 0.11 |
| *Eubacterium_nodatum_group* | 0.32 | 0.38 | 0.35 | 0.018 | 0.39 |
| *U29-B03* | 0.22^a^ | 0.38^b^ | 0.43^b^ | 0.036 | 0.03 |
| *Eubacterium_ventriosum_group* | 0.16 | 0.41 | 0.44 | 0.066 | 0.18 |
| *Ruminiclostridium* | 0.35 | 0.31 | 0.34 | 0.020 | 0.85 |
| *Family_XIII_AD3011_group* | 0.33 | 0.36 | 0.30 | 0.028 | 0.72 |
| *norank_f__Lachnospiraceae* | 0.33 | 0.30 | 0.28 | 0.020 | 0.62 |
| *Anaerovorax* | 0.32 | 0.25 | 0.28 | 0.025 | 0.58 |
| *Ruminococcaceae_gauvreauii_group* | 0.32 | 0.23 | 0.24 | 0.029 | 0.51 |
| *Selenomonas* | 0.24 | 0.30 | 0.22 | 0.049 | 0.82 |
| *Pirellula* | 0.18 | 0.26 | 0.31 | 0.022 | 0.05 |
| *Desulfovibrio* | 0.26 | 0.22 | 0.24 | 0.033 | 0.89 |
| *Pseudobutyrivibrio* | 0.37 | 0.20 | 0.15 | 0.057 | 0.26 |
| *norank_f__p-251-o5* | 0.12 | 0.30 | 0.30 | 0.039 | 0.09 |
| *Lachnospiraceae_XPB1014_group* | 0.17 | 0.26 | 0.24 | 0.027 | 0.42 |
| *Marvinbryantia* | 0.15 | 0.30 | 0.23 | 0.029 | 0.09 |
| *UCG-005* | 0.23 | 0.19 | 0.25 | 0.031 | 0.69 |
| *Lachnospiraceae_UCG-002* | 0.16 | 0.20 | 0.22 | 0.015 | 0.38 |
| *norank_f__Oscillospiraceae* | 0.19 | 0.20 | 0.17 | 0.015 | 0.83 |
| *norank_f__Christensenellaceae* | 0.17 | 0.19 | 0.20 | 0.020 | 0.89 |
| *Defluviitaleaceae_UCG-011* | 0.09 | 0.26 | 0.20 | 0.031 | 0.07 |
| *p-1088-a5_gut_group* | 0.12^a^ | 0.19^b^ | 0.18^b^ | 0.011 | 0.01 |

One-analysis of variance (ANOVA) was used to compare the means. Values within the same row with different letters are significantly different (*P* < 0.05).

Control = basal TMR ( no supplemental RP-Leu or RU-Leu); RU-Leu = basal TMR + 12 mg RU-Leu; RP-Leu = basal TMR +16 mg RP-Leu.

**Table S7**

Relative abundance (%) of the functional pathways in the 3 groups at 6 h *in vitro* (n = 5, Top 40).

| Items | Control | RU-Leu | RP-Leu | SEM | | *P*-value |
| --- | --- | --- | --- | --- | --- | --- |
| Metabolic pathways | 25.57 | 25.40 | 25.37 | 0.038 | 0.05 | |
| Biosynthesis of secondary metabolites | 13.15 | 12.94 | 12.95 | 0.043 | 0.08 | |
| Microbial metabolism in diverse environments | 5.89 | 5.89 | 5.89 | 0.010 | 0.99 | |
| Biosynthesis of amino acids | 5.83 | 5.89 | 5.91 | 0.011 | 0.05 | |
| Carbon metabolism | 4.01 | 3.96 | 3.96 | 0.017 | 0.39 | |
| Ribosome | 3.50 | 3.45 | 3.44 | 0.013 | 0.16 | |
| Purine metabolism | 2.19 | 2.18 | 2.18 | 0.003 | 0.31 | |
| ABC transporters | 1.75^a^ | 2.07^b^ | 2.09^b^ | 0.061 | 0.02 | |
| Two-component system | 1.66^a^ | 1.83^b^ | 1.84^b^ | 0.030 | 0.01 | |
| Pyrimidine metabolism | 1.74 | 1.73 | 1.73 | 0.009 | 0.92 | |
| Quorum sensing | 1.42^a^ | 1.57^b^ | 1.57^b^ | 0.028 | 0.02 | |
| Amino sugar and nucleotide sugar metabolism | 1.50 | 1.52 | 1.52 | 0.012 | 0.78 | |
| Glycolysis / Gluconeogenesis | 1.52 | 1.51 | 1.51 | 0.006 | 0.70 | |
| Aminoacyl-tRNA biosynthesis | 1.51 | 1.52 | 1.51 | 0.002 | 0.33 | |
| Oxidative phosphorylation | 1.56 | 1.46 | 1.48 | 0.026 | 0.31 | |
| Cysteine and methionine metabolism | 1.47 | 1.48 | 1.47 | 0.005 | 0.82 | |
| Glycine, serine and threonine metabolism | 1.41 | 1.39 | 1.39 | 0.003 | 0.09 | |
| Carbon fixation pathways in prokaryotes | 1.40 | 1.38 | 1.38 | 0.007 | 0.35 | |
| Pyruvate metabolism | 1.34 | 1.36 | 1.36 | 0.005 | 0.54 | |
| Homologous recombination | 1.31 | 1.30 | 1.29 | 0.003 | 0.10 | |
| Alanine, aspartate and glutamate metabolism | 1.30 | 1.30 | 1.30 | 0.006 | 0.99 | |
| 2-Oxocarboxylic acid metabolism | 1.27 | 1.23 | 1.24 | 0.009 | 0.33 | |
| Starch and sucrose metabolism | 1.17 | 1.20 | 1.21 | 0.019 | 0.72 | |
| Peptidoglycan biosynthesis | 1.18 | 1.19 | 1.18 | 0.004 | 0.65 | |
| Phenylalanine, tyrosine and tryptophan biosynthesis | 1.19 | 1.18 | 1.18 | 0.004 | 0.54 | |
| Mismatch repair | 1.10 | 1.10 | 1.10 | 0.002 | 0.92 | |
| Porphyrin and chlorophyll metabolism | 1.02^a^ | 1.10^b^ | 1.11^b^ | 0.014 | 0.01 | |
| Glyoxylate and dicarboxylate metabolism | 1.06 | 1.05 | 1.06 | 0.003 | 0.76 | |
| Pentose phosphate pathway | 1.02^a^ | 1.05^b^ | 1.05^b^ | 0.005 | 0.01 | |
| Citrate cycle (TCA cycle) | 1.01 | 0.94 | 0.93 | 0.015 | 0.06 | |
| Methane metabolism | 0.94^a^ | 0.96^b^ | 0.96^b^ | 0.003 | < 0.01 | |
| Fatty acid metabolism | 0.98 | 0.92 | 0.92 | 0.018 | 0.26 | |
| DNA replication | 0.94 | 0.92 | 0.92 | 0.003 | 0.23 | |
| Fructose and mannose metabolism | 0.91 | 0.93 | 0.94 | 0.013 | 0.63 | |
| Fatty acid biosynthesis | 0.88 | 0.83 | 0.82 | 0.014 | 0.17 | |
| Protein export | 0.85 | 0.84 | 0.84 | 0.002 | 0.06 | |
| Pantothenate and CoA biosynthesis | 0.85 | 0.83 | 0.83 | 0.002 | 0.08 | |
| RNA degradation | 0.82 | 0.82 | 0.81 | 0.002 | 0.54 | |
| Butanoate metabolism | 0.80 | 0.83 | 0.82 | 0.006 | 0.41 | |
| Carbon fixation in photosynthetic organisms | 0.81 | 0.80 | 0.80 | 0.004 | 0.35 | |

One-analysis of variance (ANOVA) was used to compare the means. Values within the same row with different letters are significantly different (*P* < 0.05).

Control = basal TMR ( no supplemental RP-Leu or RU-Leu); RU-Leu = basal TMR +12 mg RU-Leu; RP-Leu = basal TMR +16 mg RP-Leu.
